# Computational RNA secondary structure design: empirical complexity and improved methods

**DOI:** 10.1186/1471-2105-8-34

**Published:** 2007-01-31

**Authors:** Rosalía Aguirre-Hernández, Holger H Hoos, Anne Condon

**Affiliations:** 1 Institute of Applied Mathematics, University of British Columbia, Vancouver, BC V6T 1Z2, Canada; 2 Department of Computer Science, University of British Columbia, Vancouver, BC V6T 1Z4, Canada

## Abstract

**Background:**

We investigate the empirical complexity of the RNA secondary structure design problem, that is, the scaling of the typical difficulty of the design task for various classes of RNA structures as the size of the target structure is increased. The purpose of this work is to understand better the factors that make RNA structures hard to design for existing, high-performance algorithms. Such understanding provides the basis for improving the performance of one of the best algorithms for this problem, RNA-SSD, and for characterising its limitations.

**Results:**

To gain insights into the practical complexity of the problem, we present a scaling analysis on random and biologically motivated structures using an improved version of the RNA-SSD algorithm, and also the RNAinverse algorithm from the Vienna package. Since primary structure constraints are relevant for designing RNA structures, we also investigate the correlation between the number and the location of the primary structure constraints when designing structures and the performance of the RNA-SSD algorithm. The scaling analysis on random and biologically motivated structures supports the hypothesis that the running time of both algorithms scales polynomially with the size of the structure. We also found that the algorithms are in general faster when constraints are placed only on paired bases in the structure. Furthermore, we prove that, according to the standard thermodynamic model, for some structures that the RNA-SSD algorithm was unable to design, there exists no sequence whose minimum free energy structure is the target structure.

**Conclusion:**

Our analysis helps to better understand the strengths and limitations of both the RNA-SSD and RNAinverse algorithms, and suggests ways in which the performance of these algorithms can be further improved.

## 1 Background

Ribonucleic acids (RNA) are macromolecules that play fundamental roles in many biological processes, and in many cases their structure is essential for their biological function. A secondary structure for an RNA strand is simply a set of pairing interactions between bases in the strand. Each base can be paired with at most one other base. Most base-pairings occur between Watson-Crick complementary bases C and G or A and U, respectively (canonical pairs). Other pairings, such as G•U, can be found occasionally. Secondary structure determines many important aspects of RNA tertiary structure; it can, for example, be used in part to explain translational controls in mRNA [1,2] and replication controls in single-stranded RNA viruses [3].

Almost all widely used computational approaches for prediction of RNA secondary structures from single sequences are based on thermodynamic models that associate a free energy value with each possible secondary structure of a strand. The secondary structure with the lowest possible free energy value, the minimum free energy (MFE) structure, is predicted to be the most stable secondary structure for the strand. There are widely used dynamic programming algorithms that, given an RNA strand of length *n*, find in Θ(*n*^3^) time the secondary structure with the lowest free energy, from the class of pseudoknot-free secondary structures. Throughout this paper, all references to secondary structures refer to pseudoknot-free secondary structures.

### 1.1 The RNA Secondary Structure Design Problem

This work focuses on the design of RNA strands that are predicted to fold to a given MFE secondary structure, according to a standard thermodynamic model such as that of Mathews et al. [4]. This *RNA secondary structure design problem*, which can be seen as the inverse of the RNA secondary structure prediction problem, is relevant because the ability to solve it will facilitate the characterization of biological RNAs by their function and the design of new ribozymes that can be used as therapeutic agents [5]. There are also applications in nanobiotechnology in the context of building self-assembling structures from RNA molecules [6].

Dirks et al. [7] described two paradigms for designing a structure. A positive design optimizes sequence affinity for the target structure, while a negative design optimizes sequence specificity to the target structure. Sequences with high affinity have energetically favourable conformations similar to the target structure. For sequences with high specificity, structures other than the target structure are energetically less favourable. Dirks et al. [7] defined several criteria to evaluate the specificity and the affinity of a structure and found that it is desirable to achieve both, high affinity and high specificity. Another solution to the RNA secondary structure design problem is the stochastic local search algorithm provided by Hofacker et al. [8], RNAinverse, the implementation of which is included in the Vienna RNA Secondary Structure Package. A more recent stochastic local search algorithm, the RNA Secondary Structure Designer (RNA-SSD) of Andronescu et al. [9] has been shown to achieve substantially better performance on artificially designed and biological RNA structures.

The purpose of this work is to understand better the factors that render RNA structures hard to design. Such understanding provides the basis for improving the performance of RNA-SSD and for characterising its limitations. To our knowledge, it has not been determined whether there is a polynomial-time algorithm for RNA secondary structure design. Schuster et al. [10] performed experiments with the RNAinverse algorithm on few small random sequences and a simple tRNA to support the hypothesis that there is no need to search huge portions of the sequence space to find a particular structure by mutation and selection. Based on these experiments, they argue that sequences sharing the same structure are distributed randomly over sequence space and that common structures, that is, structures that have many sequences that fold into them, can be accessed from an arbitrary sequence compatible with the target structure by a number of mutations much smaller than the sequence length. These results are based on small sequences and therefore they do not give insight into the computational complexity of the design problem. On the other hand, Andronescu et al. [9] found evidence that some ribosomal RNA structures are difficult to design and that the correlation between the size and hardness is not very strong. For example, from a set of four ribosomal RNA structures of length between 260 and 299, RNA-SSD solves two structures in less than five CPU seconds on a high-performance PC, compared to three CPU minutes expected run time for the third structure and 40 CPU minutes for the fourth one. (These expected run times are determined based on 50 runs using Formula 1, which takes into account the effect of unsuccessful runs.)

Therefore, to gain insights into the practical complexity of the RNA secondary structure design problem, we present an empirical analysis of an improved version of the RNA-SSD algorithm of Andronescu et al. [9] that has been developed in the context of this work (this variant is described in Section 1.2). We also include the Vienna RNAinverse algorithm [8] in our analysis. Our analysis uses randomly generated structures, obtained by folding a randomly generated sequence with the *RNAfold *function from the Vienna Package, as well as structures that were generated according to a statistical model derived from biological RNA structures; we refer to the latter as *biologically motivated structures*. Our scaling analysis supports the hypothesis that the running time of the algorithms is polynomial in the size of the input structure. In addition, we identify structures that cannot be designed by the RNA-SSD algorithm, and in some cases show that these structures are provably undesignable, in the sense that there exist no RNA sequences with these MFE structures under the thermodynamic model by Turner et al.

Secondly, we introduce and analyse a version of RNA-SSD that additionally allows the specification of primary structure constraints. Such constraints are important, for example, when designing RNAs such as ribozymes or tRNAs, where certain base positions must be fixed in order to permit interaction with other molecules. We show that depending on their number and location, such constraints can have a significant impact (positive or negative) on the running time of the design algorithm. Our results indicate that when the primary structure constraints are restricted to stems, our new version of RNA-SSD is faster than when the constraints are distributed randomly, and in both cases the algorithm's median expected running time scales polynomially with the size of the structure to be designed.

### 1.2 The RNA-SSD algorithm

The RNA secondary structure design problem can be formalised as a discrete constraint satisfaction problem, where the constraint variables are the positions in the desired RNA strand, the values assigned to these variables correspond to the bases at the respective positions, and the constraints capture the base-pairings that define the given secondary structure. For this problem, evaluating the quality of candidate solutions is computationally expensive, since it uses an implementation of the algorithm of Zuker and Stiegler [11] for prediction of the MFE (minimum free energy) secondary structure of a given RNA sequence. Zuker's algorithm has time complexity Θ(*n*^3^), where *n *is the length of the given sequence. Unfortunately, a single local reassignment of a base in the sequence can result in a completely different MFE secondary structure. It is not known whether provably efficient (that is, polynomial-time algorithms) for the RNA secondary structure design problem exist, and previous work on solving this problem is based on heuristic approaches. Because of its relevance for this work, in the following we give a brief overview of the RNA-SSD algorithm, which is described in detail by Andronescu et al. [9].

RNA-SSD is a stochastic local search (SLS) algorithm that iteratively modifies single unpaired bases or base-pairs of a candidate strand in order to obtain a sequence that is predicted to have the target MFE structure. The *RNAfold *function from Vienna Package is used to evaluate the quality of candidate solutions, as it is the most efficient publically available implementation of Zuker's algorithm of which we are aware. Since evaluating the quality of candidate solutions is computationally expensive, RNA-SSD hierarchically decomposes the input secondary structure into small substructures. The SLS algorithm is only applied to the smallest substructures, and the corresponding partial solutions are combined into candidate solutions for larger subproblems guided by a decomposition tree. Since the smaller subproblems are not independent, this does not always result in valid designs for the corresponding larger substructure. Consequently, multiple attempts (involving additional calls to the core SLS procedure) are often required before partial solutions can be combined successfully. There are other components in the algorithm that are also important for its performance. For instance, a biased probabilistic method is used for generating a good initial design for the RNA strand that increases the chances of correct folding. This initialisation assigns bases probabilistically to the strand, using different probabilistic models for base positions that are paired and unpaired in the target structure. The algorithm also ensures that complementary stretches of bases are avoided across the design, except where desired along two sides of a stem.

## 2 Results

The main contributions of our work fall into four categories: Improvements to the RNA-SSD algorithm, including support for primary structure constraints; results on the scaling of run time for our new RNA-SSD algorithm and RNAinverse on design problems *without *primary structure constraints; results regarding the undesignability of certain structures; and results on the impact of primary structure constraints on the relative difficulty and scaling of run time for our new RNA-SSD algorithm. In the following, we describe each of these results in detail.

### 2.1 Improvement of the RNA-SSD algorithm

**Figure 1:**
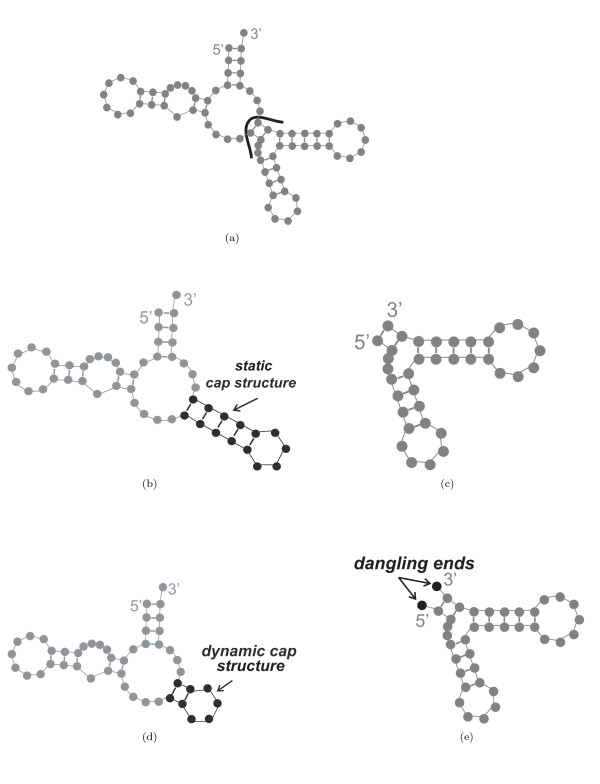
(a) Randomly generated structure of length 75 (RND-75-n62) with loops separated by short stems. The line represents the location where the structure is split into two substructures. Parts (b) and (c) show the corresponding substructures with a static cap structure and dangling ends, respectively. Parts (d) and (e) show the same substructures with a dynamic cap structure and dynamic dangling ends, respectively.

In preliminary experiments, we found that some structures are very difficult to design by using the hierarchical decomposition of Andronescu et al. [9]. This is the case, for example, for structures that have two loops separated by a very short stem (see Figure 1a). Recall that after splitting a structure (which is always done at a multiloop [9]), it is necessary to connect the two free ends created by the split such that both resulting substructures have exactly two free ends. To create structural boundary conditions at the split points that are similar to those of the original structure, this connection is achieved by merging the free ends of one fragment with those of a static *cap structure*, which is a small hairpin loop of size four (consisting of four unpaired bases and five paired bases, see Figure 1b); furthermore, two unpaired bases are added to the free ends of the other fragment if it contains a bulge directly after the first base pair. (Note that the example structure in Figure 1a does not contain bulges; therefore, no unpaired bases are added after splitting, resulting in the fragment shown in Figure 1c).

This mechanism can be improved by introducing a dynamic cap structure and dynamic dangling ends in order to create structural boundary conditions that are exactly identical to the original structure in terms of the number of paired and unpaired bases adjacent to the split point. In our new mechanism, the number of paired bases in the cap structure added to one fragment depends on the number of paired bases at the beginning of the other substructure (Figure 1d). Furthermore, we add one unpaired base to the 5' (3') end of a substructure if, and only if, its adjacent base in the original structure is a free base (Figure 1e). It is sufficient to add one unpaired base to the end of the structure since dangling ends of more than one base do not contribute to the free energy of the structure [4]. The use of dynamic instead of static cap structures and free ends often results in improved performance for structures that are hard to design. For instance, the expected time to design the structure Rand-75-n62 from Figure 1a is reduced from 88446.79 CPU seconds to 26.99 CPU seconds when using the new dynamic mechanism. (All run times were measured on a reference machine using an Intel Xeon 2.4 GHz CPU, which is specified in more detail in Section 5.) More extensive testing on sets of random (RND-75), biologically motivated (BIOM-200) and biological structures (see Section 5) have shown that the average run time of the new version of RNA-SSD is very close to that of the old version on our set of biological structures, but about 5 times lower for the random structures and 16 times lower for the biologically motivated structures. More detailed analysis shows that the use of the new dynamic mechanism leads to run time reductions of up to four orders of magnitude on about half of the structures. However, in many other cases run time is somewhat increased, and sometimes, more severe performance degradation is observed.

We also extended RNA-SSD to support primary structure constraints, that is, constraints on the bases that occur in certain sequence positions. The additional sequence constraints limit certain sequence positions to specific bases or sets of bases. For this purpose, the standard IUPAC symbols [12] listed in Table 1 are supported. Primary structure constraints facilitate the design of more realistic structures. For instance, when a ribozyme is re-engineered to make it more stable, certain bases of the molecule can be modified while others are constrained because they define the cleavage site [13]. Sequence constraints are also needed in the design of nanostructure components with "sticky ends" [14,15].

**Table 1:** IUPAC nomenclature for nucleic acids.

Symbol	Meaning	Origin of designation
G	G	Guanine
A	A	Adenine
T	T	Thymine
C	C	Cytosine
R	G or A	puRine
Y	T or C	pYrimidine
M	A or C	aMino
K	G or T	Ketone
S	G or C	Strong interaction (3 H bonds)
W	A or T	Weak interaction (2 H bonds)
H	A or C or T	not-G, H follows G in the alphabet
B	G or T or C	not- A, B follows A
V	G or C or A	not-T (not-U), V follows U
D	G or A or T	not-C, D follows C
N	G or A or T or C	aNy

Our extended version of RNA-SSD supports primary structure constraints as follows. Given a sequence specification using the IUPAC symbols listed in Table 1, first, all pairs of constrained bases are checked for feasibility, that is, for whether there are Watson-Crick-complementary bases or wobble pairs that satisfy the given primary structure constraints. Then, base constraints are propagated across all paired positions, that is, the set of allowed bases for each paired sequence position is adjusted to take into account constraints on the other base involved in the pairing. This improves the efficiency of the subsequent search process by restricting the number of bases that have to be potentially considered for the respective sequence positions. When initialising the search process, we ensure that all bases are consistent with the given primary structure constraints (after propagation). Furthermore, whenever we modify a base assigned to a sequence position during the search, we ensure that the respective primary structure constraints (if any) remain satisfied; in other words, we ensure that at all times during the search process, all primary structure constraints are satisfied.

### 2.2 Analysis of RNA-SSD and RNAinverse on secondary structures without constraints

We now report results from our analysis of the empirical complexity of solving RNA secondary structure design problems with the improved version of RNA-SSD and with the RNAinverse algorithm. We performed experiments on random and biologically motivated structures of different lengths. (Details of our experimental protocol are given in the Section 5.)

We study the behaviour of the algorithm on biological structures since it will have an impact in biological applications such as ribozyme design. Because of the limited availability of true biological structures, we generated structures with biological characteristics based on the set of real structures listed in Table 2. The statistics reported in Table 3 summarise salient structural properties of these naturally occurring RNAs. We used a probabilistic model based on this data to generate new sets of RNA structures with similar properties. This allows us to evaluate RNA-SSD (and RNAinverse) on a large number of structures and helps us to reduce the chance of drawing erroneous conclusions from a small set of atypical results. Using this approach, we can also control salient properties of the generated structures, such as the size of a given structure, the relative prevalence of bulges or the average branching of multiloops, and study their impact on the performance of RNA design algorithms.

**Table 2:** Biological RNA structures.

No.	Description	Size (bases)
1	Minimal catalytic domains of the hairpin ribozyme satellite RNA of the Tobacco ringspot virus	65
2	U3 snoRNA 5' domain from *Chlamydomonas reinhardtii, in vivo *probing	79
3	*H. marismortui *5 S rRNA	122
4	VS ribozyme from Neurospora mitochondria	167
5	R180 ribozyme	178
6*	XS1 ribozyme, *Bacillus subtilus *P RNA based ribozyme	314
7*	Homo Sapiens RiboNuclease P RNA	342
8	S20 mRNA from *E. coli*	372
9	*Halobacterium cutirubrum *RNAse P RNA	375
10	Group II intron ribozyme D135 from *Saccharomyces cerevisiae *mitochondria	583

Biological structures obtained from the literature and used by Andronescu et al. [9]. The structures marked with an asterisk (*) were obtained from original, pseudoknotted structures by eliminating 8 base pairs in each case to remove the pseudoknot.

**Table 3:** Statistics of biological structures from Table 2.

	Hairpins	Stems	2-Branch loops	Multiloops	Bulges
Size	[4,8]	[3,12]	[4, 11]	[6,17]	[1,3]
Number	-	-	[1,8]	[0,5]	[0,0.17]*
Branches	-	-	-	[3,4]	-

Properties of the structures from Table 2; the intervals specify the minimal and maximal values observed for the respective features. These parameters were used to generate structures with biological properties. * This value denotes the ratio of bulges to base pairs in the stems.

**Figure 2:**
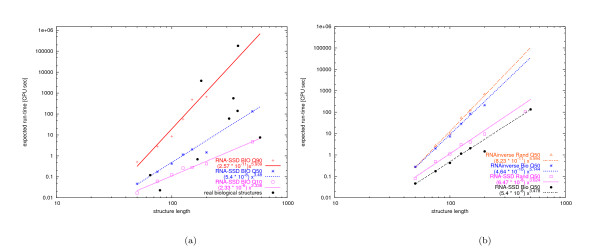
**Scaling analysis of RNA-SSD and RNAinverse**. Scaling analysis of the expected run time (y-axis) of structures of lengths 50, 75, 100, 125, 150, 200 and 450 (x-axis). A logarithmic scale is used on both axes. The lines correspond to best fits of the data, for structures with lengths 50 to 150, using a polynomial that is specified in each case. The expected run time for structures longer than 150 appear close to the corresponding fit line. (a) Expected run time of RNA-SSD to design biological structures and median (Q50), 0.1-quantile (Q10) and 0.9-quantile (Q90) of expected run time for RNA-SSD applied to biologically motivated structures. (b) Median of expected run time of random and biologically motivated structures using RNA-SSD and RNAinverse. The structures of length 200 are the largest structures from the respective data sets that we designed with RNAinverse.

Figure 2a shows the median expected run time for different structure lengths (where the median is over the structures in a set and the expectation is over multiple runs of the algorithm on a given structure), as well as the expected run time for the structure at the 10% and the 90% quantile for the biologically motivated structures. We also show the expected run times for the set of real biological structures summarised in Table 2. Notice that the empirical complexity for designing these real structures fits well within the range of complexity observed for our biologically motivated sets of structures, which provides some evidence that the probabilistic model underlying these sets is reasonably plausible for the purposes of this study. Furthermore, the data in Figure 2a indicate that the expected run time of RNA-SSD scales polynomially with structure size for median difficulty as well as for the 10% and 90% quantiles of these structure distributions, where the degree of the polynomial is higher for higher quantiles.

**Figure 3:**
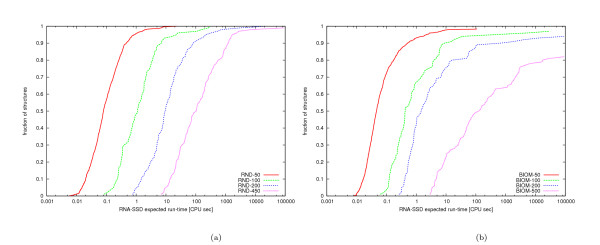
**Search cost distribution of RNA-SSD**. Distribution of expected run time of RNA-SSD on (a) random structures and (b) biologically motivated structures. For each point, the x-value indicates an expected run time and the y-value corresponds to the fraction of structures whose run time is at most the x-value. We arbitrarily (but unambiguously) report the expected run time for structures that RNA-SSD is unable to design as 10^6 ^CPU seconds.

**Figure 4:**
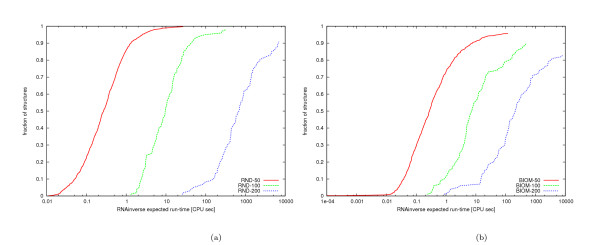
**Search cost distribution of RNAinverse**. Distribution of expected run time of RNAinverse on (a) random structures and (b) biologically motivated structures. We report the expected run time for structures that RNAinverse is unable to design as 10^6 ^CPU seconds.

As can be seen from Figure 2b, we obtained similar results for random structures as well as when using RNAinverse. Notice that overall, RNA-SSD performs substantially better than RNAinverse, and that random structures tend to be somewhat more difficult to design than biologically motivated structures. Distributions of expected run time for RNA-SSD over our sets of random and biologically motivated structures of various sizes are shown in Figures 3 and 4. Note that there is a large variation in difficulty between structures from the same set. Also, there are some structures that RNA-SSD is unable to design (the same holds for RNAinverse, as will be explained later).

**Figure 5:**
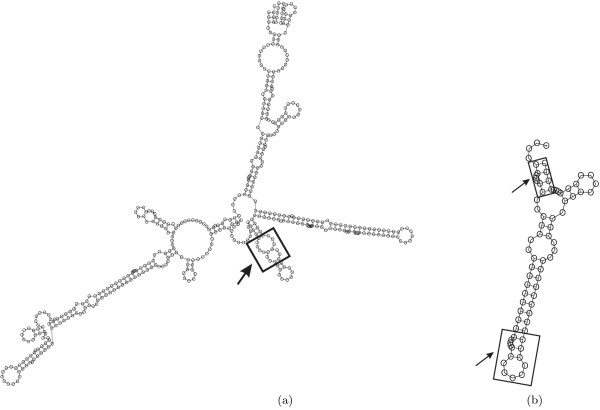
**Examples of structures not designed by RNA-SSD**. Structures not designed by RNA-SSD have short stems separated by loops, indicated by arrows in the Figure. (a) Random structure of length 450 (RND-450-n84). This is the only random structure in our data set that RNA-SSD did not design. Note that it has two internal loops separated only by one base pair. (b) Biologically motivated structure of length 74 (BIOM-50-n262).

The random structures are designable by construction since they were obtained by folding a set of random sequences with the *RNAfold *function from the Vienna package (see Section 5). RNA-SSD was able to design all of these structures except one of length 450 (Figure 3a). This structure has several short stems separated by loops (Figure 5a). In particular, it has two internal loops next to each other; one of these is slightly asymmetric with seven unpaired bases, while the other is symmetric with four unpaired bases. Although allowed by the thermodynamic model, this motif is hard to design. (This will be discussed in more detail in the later section on undesignable structures.) RNAinverse failed to design 1.16% (i.e., 28/2400) of the random structures of length 200 or less and was not evaluated on larger structures because of excessive run time requirements.

As can be seen from Figure 3b, there are biologically motivated structures of every length that RNA-SSD was unable to design; none of these structures could be designed by RNAinverse, which also did not succeed to design a number of other structures. Overall, 6.83% and 2.21% (i.e., 164/2400 and 53/2400) of the biologically motivated structures could not be designed by RNAinverse and RNA-SSD, respectively; this indicates that biologically motivated structures are more difficult to design with these algorithms than randomly generated structures.

To further explore RNA-SSD's ability to design larger structures, we evaluated its performance on two additional sets, containing random structures of length 450 and biologically motivated structures of length 500, respectively. In these experiments, we found that RNA-SSD designed 99.78% of the randomly generated structures and 94.4% of the biologically motivated ones within a cutoff time of 30 CPU minutes.

### 2.3 Undesignable structures

**Figure 6:**
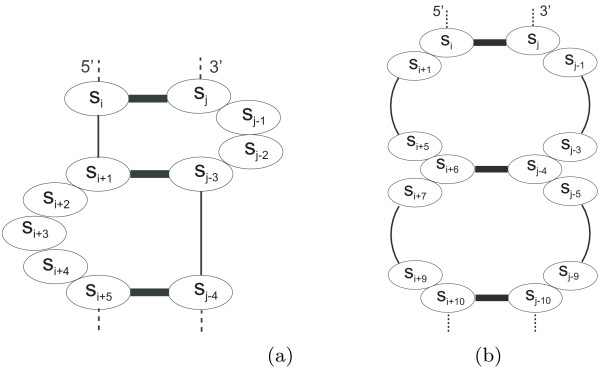
**Undesignable motifs**. Two structure motifs of our data set that are not compatible with the thermodynamic model. Bold lines represent base pairs. (a) Motif B: bulges separated by one base pair. (b) Motif 2I: internal loops separated by one base pair.

When examining the structures that appeared to be undesignable by the RNA-SSD algorithm, we found that they typically have short stems separated by loops, as shown in Figure 5. Further analysis revealed two kinds of motifs that are impossible to design for any algorithm based on the current thermodynamic model. One of these consists of two bulges next to each other, separated only by one base pair (see Figure 6a); the other one is formed by two internal loops, separated also by a single base pair (Figure 6b). For internal loops of size bigger than four and for asymmetric internal loops of size four, these motifs are predicted to be unstable by the current thermodynamic model, and are hence undesignable (a formal proof is given in the appendix). However, it is possible to design two internal loops separated by one base pair if at least one of them is symmetric and has four unpaired bases.

Unstable motifs were found in several biologically motivated structures, and they also seem to appear in nature. For example, according to the Comparative RNA Web (CRW) Site, which provides RNA secondary structures based on comparative sequence analysis [16,17], the small subunit ribosomal RNA of *Acanthamoeba castellanii *[16,17] has three adjacent bulges of size two, one and three, respectively (these bulges are located in positions 1578–1583 and 1841–1848). Similarly, the CRW structure for the small subunit ribosomal RNA of *Escherichia coli *[16-18] has two adjacent internal loops of size seven and nine, respectively (this motif is found in positions 1963–1972 and 1994–2005).

### 2.4 Analysis of RNA-SSD on secondary structures with constraints

From the previous experiments we learned that the empirical time-complexity of the RNA design problem is polynomial for random and biologically motivated structures. Next, we will investigate the hardness of the problem when primary structure constraints are imposed on the design of the biologically motivated structures that we used for the unconstrained case.

The hardness of an instance of this constrained secondary structure design problem not only depends on the given secondary structure, but also on the set of primary structure constraints. To capture the impact of the primary structure constraints on the performance of RNA-SSD, we used every secondary structure with a number of different sets of primary structure constraints; furthermore, because of the stochastic nature of RNA-SSD, we performed multiple runs of our algorithm for each such problem instance. The expected CPU time required to design a structure with a given set of primary structure constraints was estimated from these runs. Most of our analysis is based on the median expected run time of RNA-SSD over all sets of constraints for a given structure. Because of the computational burden incurred by the large number of runs per secondary structure required by this protocol, we performed these experiments on smaller sets of biologically motivated structures; these sets were obtained by uniform random sampling (without replacement) from the respective sets used for our empirical analysis of the unconstrained case. Two different methods were used to create sets of primary structure constraints. One of these essentially selects the base positions to be fixed within the given structure at random, while the other fixes the base assignments of entire stems. In both cases, the bases in the selected positions are fixed according to a sequence that folds stably into the given structure. (Details are described in Section 5.)

**Figure 7:**
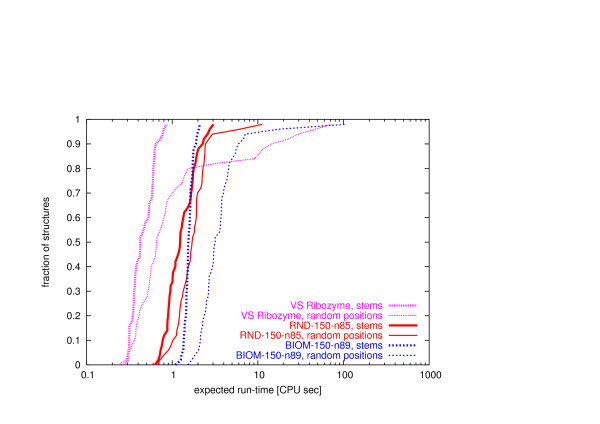
**Search cost distribution for the design of structures with primary structure constraints using RNA-SSD**. Distribution of expected run time of RNA-SSD on three structures of approximately 150 bases: RND-150-n85, BIOM-150-n89 and VS ribozyme from Neurospora mitochondria. The structures were designed with two sets of primary base constraints: one where the bases are fixed at random positions and another where the bases are fixed on stems for each structure. Both sets have the same range [*a, b*] of constrained bases after propagation, where *a *and *b *are smallest and largest number of bases constrained if 50% of stems are fixed in a given structure. We fixed 50% plus one stem when a structure had an odd number of stems.

As can be seen in Figure 7, the hardness of a constrained design problem varies significantly depending on the given set of constrained bases. In particular, constraining entire stems rather than randomly selected bases tends to result in slightly easier problems.

**Figure 8:**
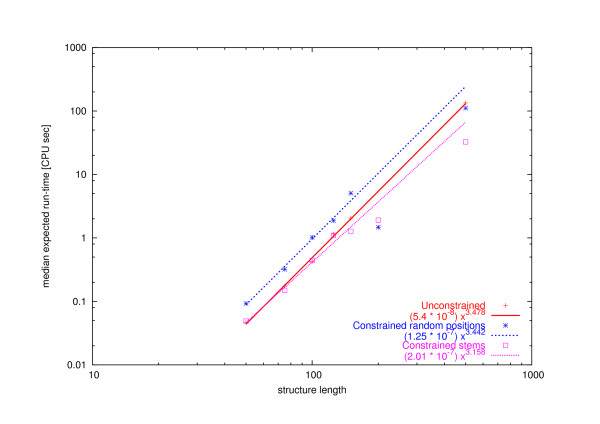
**Scaling analysis on biologically motivated structures with different primary structure constraints using RNA-SSD**. Scaling analysis for the median expected run time of biologically motivated structures with no primary base constraints and with bases constrained in fifty percent of random positions and fifty percent of stems. The lines represent the polynomial that best fits the data for structures with lengths 50, 75 and 100. The experiment with primary structure constraints is computationally expensive, and for this reason, fewer structures of each length were used. Note that the run times for constrained structures longer than 100 appear below the corresponding fit line.

Figure 8 illustrates the scaling of search cost for solving our sets of biologically motivated RNA secondary structure design problems with different types of primary structure constraints compared to the unconstrained case. Our empirical results indicate that the median expected run time scales polynomially with the size of the structures for the unconstrained case and for constraints located in random positions or in stems; in all three cases the median run time is approximated by a polynomial of degree close to three. There is some indication that in the case of constrained stems (which play a major role in stabilising RNA secondary structure) better scaling behaviour is observed than for base constraints in randomly chosen positions or no base constraints at all.

### 2.5 Performance of RNA-SSD with different number and locations of primary base constraints

In a second series of experiments, we studied the correlation between the number of bases constrained and the performance of the RNA-SSD algorithm. The experiments were conducted using some biological structures from Table 2 as well as biologically motivated structures. Table 4 shows some features of these structures. The two biological structures chosen for this experiment are the *VS ribozyme from Neurospora mitochondria *and the *group II intron ribozyme D135 from Saccharomyces mitochondria*. The biologically motivated structures were chosen according to various criteria. *Bio-150-nl4 *has the same size and number of multiloops as the VS ribozyme; for *Bio-150-n38*, the run time required by RNA-SSD (without primary structure constraints) to design the structure is very similar to that required by VS ribozyme; *Bio-200-nl9 *is a longer structure that has several multiloops, like the group II intron ribozyme; and *Bio-150-nl2 *is particularly easy to design. In every case, the primary structure constraints are based on sequences that are computationally predicted to fold into the target structure (see Section 5 for more details); therefore, the respective design problems are solvable by construction.

**Table 4:** Structures for the study of the performance of RNA-SSD as a function of primary structure constraints.

No.	Description (source)	Size (bases)	expected run time [CPU sec]	number of multiloops	number of of stems
1	VS ribozyme from Neurospora mitochondria	167	0.64	2	11
2	Bio-150-n38	172	0.53	1	9
3	Bio-150-nl4	167	12.94	2	10
4	Group II intron ribozyme D135 from *Saccharomyces cerevisiae *mitochondria	584	11.54	5	32
5	Bio-200-nl9	208	7.62	3	12
6	Bio-150-nl2	150	0.16		6

Set of structures used to study the correlation between the primary structure constraints and the performance of RNA-SSD. Structures with similar characteristics (such as size, number of multiloops, etc.) appear in the same group. The structure Bio-150-nl2 was included in this set because it is relatively easy to design.

**Figure 9:**
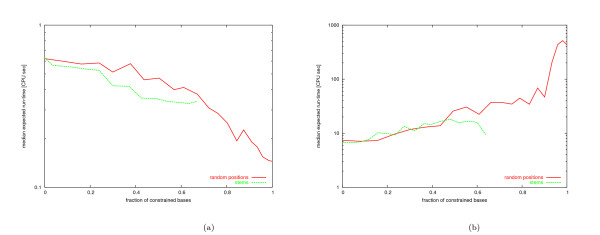
**Impact of constrained bases on the difficulty of secondary structure design using RNA-SSD**. Correlation between the fraction of bases constrained in a particular structure (x-axis) and the median expected run time for designing the structure with RNA-SSD (y-axis). We report the fraction of constrained bases after propagation for constraints on randomly chosen base positions. This fraction, for both randomly chosen bases and stems, corresponds to the median fraction of bases constrained in a set of 50 constraints that were generated by fixing a given percentage of bases or stems. There are two curves in each graph, one for designing structures with base constraints located in random positions and the other for constraints located in stems. (a) VS ribozyme from Neurospora mitochondria; (b) Group II intron ribozyme D135 from *Saccharomyces*.

Figure 9 shows how the hardness of the design problem depends on the fraction of constrained bases for randomly located base constraints and for constrained stems. As can be seen from these results, there are some cases in which base constraints of either type render a secondary design problem easier, while in other cases, we observe a substantial increase in hardness as a critical number of bases is constrained.

## 3 Discussion

In earlier work by Andronescu et al. [9], no clear correlation has been detected between the size of a given RNA structure and the performance of RNA secondary structure design algorithms such as RNA-SSD. Here, we used a bigger set of structures to investigate the empirical complexity of RNA design and found a clear correlation between the size of the structure and the performance of the two algorithms we studied, RNA-SSD and RNAinverse. In particular, the results of our empirical scaling analysis for the unconstrained RNA secondary structure design problem indicates that the expected run time of RNA-SSD and RNAinverse increases polynomially with the size of the structure to be designed. However, RNA-SSD shows substantially better scaling behaviour than RNAinverse, as indicated by a significant difference in the degree of the polynomial obtained when approximating the scaling with expected run time with structure size (in bases).

Both, RNAinverse and RNA-SSD, failed to design some structures, but there was no case in which RNA-SSD was unable to design a structure solved by RNAinverse. Some of the structures that could not be designed by RNA-SSD contain motifs that are provably not allowed by the thermodynamic model of RNA secondary structure and are hence inherently undesignable using that model. Such motifs contain short stems that are not stable enough to compensate for the penalty associated with the adjacent loops; we have observed similar motifs in all structures that RNA-SSD failed to design, and suspect that most (if not all) of these structures may be inherently undesignable. On the other hand, we also found inherently undesignable structural motifs in trusted structures of biological RNAs. This could be due to inaccuracies of the thermodynamic model commonly used for RNA secondary structure, tertiary structure effects or interaction of the RNA with other molecules, which prevent it from folding into its "true" MFE conformation.

We also found that artificially generated structures with statistical features derived from trusted biological structures (here called "biologically motivated structures") are easier to design than structures of random sequences, probably because they contain more structural motifs that are easy to design. Also, for the undesigned trusted biological structures, it is not clear *a priori *whether they can be designed using the standard thermodynamic model. The fact that the empirical hardness of designing trusted biological structures is very similar to that of designing our biologically motivated structures provides evidence that the latter capture important structural features of real RNAs and thus provide a good test-bed for studying RNA secondary structure design.

One of the improvements over the first version of our RNA-SSD algorithm (as described by Andronescu et al. [9]) introduced in this work is our use of dynamic cap structures and dangling ends to better approximate the boundary conditions encountered at the split points used during hierarchical decomposition of a given RNA secondary structure into substructures. This modification can lead to a significantly increased chance to obtain a sequence that folds into the desired structure when merging the subsequences designed for the respective substructures; this is particularly advantageous in the design of difficult structures like the one in Figure 1a. We did not find evidence that this new version of RNA-SSD can design structures that the previous version cannot handle, given sufficiently long running times.

However, we observed marked improvements in the running time and success rates in many cases. For example, Andronescu et al. [9] found a particular ribosomal RNA structure obtained from the Ribosomal Database Project, *Leptospira interrogans strain 94–7997013 *to be particularly hard to design even though it has only 289 bases. This RNA contains a structural motif consisting of two internal loops separated by one base pair, similar (but not identical) to the undesignable motif shown in Figure 6b. They reported an expected run time of 2517.57 CPU seconds to design this structure with a success rate of six in fifty runs when using a cutoff time of 3600 CPU seconds. Using our improved version of RNA-SSD, the expected time to solve this structure is 1170.23 CPU seconds with a success rate of forty four in fifty runs using the same machine and cutoff time. This structure is hard to design because it contains two internal loops separated by a single base pair only; one of these is a symmetric internal loop of size four and the other is slightly asymmetrical with size five. By introducing an additional base pair between these internal loops, the structure becomes much easier to design, requiring of our new version of RNA-SSD only an expected run time of 415.89 CPU seconds with a success rate of forty nine in fifty runs.

**Figure 10:**
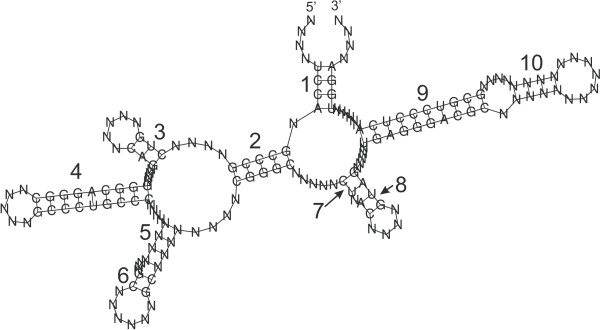
**Biologically motivated structure Bio-150-n14**. Biologically motivated structure with ten stems. When constraining the bases in stems 7 and 8, this structure is hard to design. The structure motif formed by these stems, which are short and separated by a bulge, is unstable.

Our study also sheds light on the hardness of designing structures with primary structure constraints. In particular, our detailed analysis of primary structure (that is, base sequence) constraints on the performance of RNA-SSD suggests that it is generally easier to design a structure when the stems are constrained. This is intuitively plausible, given that generally, stems represent the most stable parts of RNA secondary structures. However, there are exceptions: structure *Bio-150-nl4 *(shown in Figure 10) was found to be difficult when more than seven stems of its ten stems were constrained. When we further analyzed the correlation between the constrained stems and the expected run time required for designing this structure, we found that constraining two particular stems, labelled 7 and 8 in Figure 10, made the design problem significantly more difficult. These short stems are separated by a bulge of size one, and they are not stable enough to compensate for the penalty incurred by the bulge. Figure 10 shows a difficult problem instance where these two stems are constrained. The structure is easy to design if the base pairs A•U in stems 7 and 8 are replaced by C•G, which is a more stable interaction.

We also observed that for structures with similar characteristics (same number of bases, multiloops or stems, or same difficulty to design without constraints), the behaviour of RNA-SSD algorithm shows significant qualitative variation. Structures such as that of the *VS ribozyme from Neurospora mitochondria *(Figure 9a) and *Bio-200-nl9 *are easier to design when the number of constrained bases increases; the same holds for structure *Bio-150-n38 *when the constrained bases are located within stems. In these cases, the base constraints reduce the size of the search space and additionally may help to steer the search process towards solution sequences.

However, the problem can also get harder as the number of constrained bases increases and then becomes easier again, as approximately 80% or more of the bases are constrained or when all the stems are constrained. This is observed for structures *Bio-150-n38, Bio-nl50-nl4 *and *Bio-150-nl2 *when using random base constraints, as well as for *Bio-150-nl4 *and the *group II intron ribozyme D135 from Saccharomyces mitochondria *when the stems are constrained (Figure 9b). In these cases, the reduction in the size of the search space caused by the base constraints is counteracted up to a point by factors that make finding solutions within these smaller spaces harder. One such factor is solution density, which can be substantially reduced by adding base constraints. Beyond a certain number of primary structure constraints, the advantages from reduction in search space size outweigh these factors, such that the problem becomes easier again. This is not surprising, since the design problem becomes trivial in the extreme case in which all base positions are constrained.

Somewhat surprisingly, as can be observed for structure *Bio-150-nl2*, there are cases where constraining all stems, leaving only unpaired base positions to be assigned by the algorithm, renders the design problem harder than constraining a smaller number of stems (data not shown). Another extreme example is the structure of the *group II intron ribozyme D135 from Saccharomyces mitochondria*, where the run time of RNA-SSD increases well beyond the point where 80% of the base positions are constrained (see Figure 9b). More detailed analysis indicates that this behaviour is caused by substructures obtained in RND-SSD's decomposition process that consist entirely of constrained bases, with the exception of the cap structure that is added to ensure appropriate boundary conditions. In some cases, these substructures are hard to design, since the correct structure is only obtained for a particular base assignment to the cap structure, which may differ from the assignment of the corresponding bases on the other side of the split point, leading to a large number of failed attempts of preserving the designed substructures when merging the subsequences. One obvious solution to this problem is to prevent structural splits that give rise to fully constrained substructures.

It should be noted that our empirical complexity results do not rule out the possibility that the RNA secondary structure design problem (with or without primary structure constraints) could be NP-hard, but suggest that such worst-case asymptotic scaling is not reflected in the typical behaviour of existing algorithms applied to distributions of random and biologically plausible structures studied here. However, careful examination of our scaling data indicates that the degree of the polynomial characterising the scaling of run time with structure size is considerably higher for the hardest structures in our test-sets than it is for typical or easy structures, which could be seen as an indication of possible exponential scaling of the run time of RNAinverse and RNA-SSD in the worst case.

## 4 Conclusion

We have introduced an empirical analysis for the design of RNA secondary structures with the RNAinverse algorithm from the Vienna RNA Package and with an improved version of RNA-SSD that supports primary structure constraints. Our analysis helps us to better understand the strengths and limitations of both algorithms. For this study we used a big set of structures (5000 in total) of different lengths generated randomly and also generated with structural and statistical properties (such as loop size, number of multiloops, etc.) based on different classes of biological RNAs. We investigate the hardness of the design of these structures without primary structure constraints and with different number and locations of base constraints in the structure. In every case the problem scales polynomially with the size of the structure. Experiments on biologically motivated structures show that in general there is an advantage in the design if we impose primary base constraints in stems. When we tried to determine if the structure design is easier as we increase the number of fixed positions, we found that this is not always the case. The design of some structures gets harder when approximately 50% of the bases are constrained. This suggests a reduction in the effective search space size that depends on the properties of the structure.

RNA-SSD performs substantially better than RNAinverse, both in terms of speed as well as with respect to the structures that can be designed within a given amount of time. We compared both algorithms on random structures without primary structure constraints and found that the scaling of the median expected run-time is about *O*(*n*^3^) for RNA-SSD and about *O*(*n*^5^) for RNAinverse, where *n *is the size of the structure. The structures not designed by RNA-SSD were also not designed by RNAinverse. Furthermore, we believe that most of these structures are undesignable because they contain motifs like the ones shown in Figure 6. In the appendix we prove that some motifs with two internal loops or two bulges that are separated by one base pair are impossible to design using the current thermodynamic model for RNA secondary structure.

We also identified some structural motifs that make the RNA design task harder (data not shown). In particular, short stems separated by loops are difficult to design. Short stems are not stable enough to compensate for the penalties associated with adjacent loops, and therefore, energetically more favourable motifs are preferred. Some of these motifs are not allowed by the thermodynamic model [4], yet they are found in biological structures. For other motifs, which have short stems separated by loops that are allowed by the thermodynamic model, it was possible to improve the performance of RNA-SSD by modifying the structural decomposition approach in such a way that at the split points, the boundary conditions from the original structure are replicated. Intuitively, this leads to an increased probability that when merging the respective subsequences, the correct secondary structure is obtained.

The results of this study suggest further improvements to the RNA-SSD algorithm. For example, it is possible that structural splitting leads to substructures that, apart from the cap structure, are completely determined by primary base constraints. Such substructures can cause artificial challenges to our search algorithm and should be treated differently. Alternatively, the structural decomposition approach could be modified in such a way that the fraction of constrained bases in each substructure is balanced. Another improvement which has already been proposed by Andronescu et al. [9] is to consider split points at motifs other than multiloops; it may be noted that such a modification could easily be extended with primary base constraints.

Interactions between RNA molecules are of substantial biological interest, and we are therefore planning to extend RNA-SSD to the design of duplexes of interacting RNAs. With this extension of the algorithm, it will be possible to design pairs of strands in biomolecular nanostructures [6] as well as ribozymes that interact with an RNA target [13]. In order to design for interaction, it is important to have a method to predict the secondary structure of two interacting RNA strands. When only pseudoknot-free duplex structures are considered, we can use the PairFold software of Andronescu et al. [19]. Pseudoknot-free interacting structures arise, for example, in the interaction of a ribozyme with its target [20]. For more complicated, pseudoknotted structures, the methods of Dirks et al. [21] or Alkan et al. [22] could be used. Another important factor in designing RNA molecules is the stability of the desired respective structure. Typically, there are many RNA sequences that can fold into a given structure, but in many cases, we are interested in finding a sequence with the most thermodynamically stable MFE target structure. Currently, RNA-SSD does not explicitly evaluate or optimise the thermodynamic stability of the desired secondary structure that is achieved by the designed sequence. In future work, we will extend RNA-SSD to support the design of stable structures based on some of the positive and negative design criteria defined by Dirks et al. [7].

Very recently, Busch and Backofen [23] have introduced a new SLS algorithm for the RNA secondary structure design problem, dubbed INFO-RNA (INnverse FOlding of RNA). Different from RNA-SSD, INFO-RNA uses dynamic programming to determine an initial sequence that adopts the given target structure *T *with the lowest possible energy. Then, it uses an improved SLS procedure that performs search steps based on a look-ahead mechanism for determining energetically favorable sequences in combination with the structural decomposition approach of RNAinverse in order to find a sequence with MFE structure *T*. In most cases, INFO-RNA performs better than the improved version of RNA-SSD described in this paper, and we therefore expect that its empirical median expected run time also shows polynomial scaling with input size (possibly with better constants than RNA-SSD).

However, compared to RNAinverse and RNA-SSD, RNA-INFO is more biased towards sequences that form low-energy structures and can hence be expected to find more restricted ensembles of solutions to any given RNA secondary structure design problem. We conjecture that by combining features of RNA-SSD and RNA-INFO, in particular RNA-SSD's less biased initialisation and balanced hierarchical decomposition approach with RNA-INFO's more efficient SLS procedure, further performance improvements could be achieved. Furthermore, RNA-INFO currently does not support primary structure constraints, and it would be interesting (and not too hard) to incorporate these into a future version.

## 5 Methods

To investigate the empirical complexity of designing structures without constraints we used the following data sets. We generated random structures by folding, with the *RNAfold *function from the Vienna package, a set of randomly generated sequences with a uniform distribution of nucleotides (see Table 5). Structures with biological characteristics were generated with the help of an RNA structure generator [9] that allows us to directly control salient properties of the structures being generated, including the overall size as well as the number and size of bulge, internal, and multiloops, and the length of stems. In order to determine these properties, we selected from the biological literature ten structures that are consistent with experimental evidence and empirical data, ranging from 60 to 600 bases in length (see Table 2). Average values of each of the features captured in the parameters of the RNA structure generator over our set of structures were used to roughly summarise the structural properties of naturally occurring RNAs (see Table 3). Using the RNA structure generator with these parameter values, several sets of biologically motivated structures were generated (see Table 6).

**Table 5:** Sets of randomly generated structures.

Set name	Size (bases)	Number of structures
RND-50	50	1000
RND-75	75	1000
RND-100	100	100
RND-125	125	100
RND-150	150	100
RND-200	200	100
RND-450	450	100

Sets of structures generated by folding random sequences with the *RNAfold *function from the Vienna RNA package. Nucleotides in the sequence are assigned uniformly at random. The sets of structures of longer than 75 bases are smaller because of the amount of time required for designing these structures.

**Table 6:** Sets of biologically motivated structures.

Set name	Size (bases)	Number of structures
BIOM-50	[50,75)	1000
BIOM-75	[75,100)	1000
BIOM-100	[100,125)	100
BIOM-125	[125,150)	100
BIOM-150	[150,175)	100
BIOM-200	[200,225)	100
BIOM-500	[500,525)	100

Sets of structures generated with the RNA structure generator, using the parameters from Table 3.

For the experiment in which RNA-SSD was used to design structures with primary structure constraints, we utilised only biologically motivated structures. This experiment was computationally expensive because it required the design of a given structure with several constraints. For this reason, we chose subsets of the previously described sets of biologically motivated structures by means of random sampling (without replacement). These subsets consist of 50 structures of the data sets BIOM-50 and BIOM-75; 45 structures of the data set BIOM-100; and 10 structures of the data sets BIOM-125, BIOM-150, BIOM-200 and BIOM-500, respectively.

The primary base constraints were generated in the following way. For each structure, we used RNA-SSD to obtain 100 sequences that are computationally predicted to fold into it. Of these, we selected the sequence that gave the most stable MFE structure and used it for generating base constraints for certain positions using two different methods. In one method, we sampled 50% of the sequence positions uniformly at random (without replacement). Additionally, when generating a constraint for a paired base, we also generated a constraint for the base to which it is paired to be fixed to the correct Watson-Crick complementary base; consequently, more than 50% of the bases may be fixed in the resulting design problem. In the other method, we sampled 50% of the stems in the given structure uniformly at random (without replacement) and fixed all bases occurring in these stems.

To control for the variation in run time of the design algorithms due to the choice of constrained bases, we generated all of the possible sets of constraints in cases where this number was found to be less than 50, and random samples of size 50 otherwise. Thus, for each structure in a test set, we considered up to 50 possible sets of constraints obtained by each of the two generation methods. For structures of length 500, which are computationally expensive to design, we used only 10 instead of 50 constraint sets (also obtained by random sampling without replacement).

All computational experiments were carried out on PCs with dual Intel Xeon 2.40 GHz processors (only one processor was used in our experiments), 512 KB cache, and 1 GB RAM running Red Hat Linux, kernel version 2.6.5-1.358smp. Both, RNA-SSD and RNAinverse are highly stochastic algorithms: when applied to the same structure multiple times, the time for finding a solution may vary substantially. (Note, however, that by using the same random seed, any run of RNA-SSD can be perfectly reproduced.) Therefore, it is necessary to perform sufficiently many runs on each problem instance in order to get reasonably stable statistics on run time. For the unconstrained experiment we performed 50 runs on a given structure and estimate the expected time required for finding a solution as

Es+(1fs−1)Eu     (1)
 MathType@MTEF@5@5@+=feaafiart1ev1aaatCvAUfKttLearuWrP9MDH5MBPbIqV92AaeXatLxBI9gBaebbnrfifHhDYfgasaacH8akY=wiFfYdH8Gipec8Eeeu0xXdbba9frFj0=OqFfea0dXdd9vqai=hGuQ8kuc9pgc9s8qqaq=dirpe0xb9q8qiLsFr0=vr0=vr0dc8meaabaqaciaacaGaaeqabaqabeGadaaakeaacqWGfbqrdaWgaaWcbaGaem4CamhabeaakiabgUcaRmaabmaabaWaaSaaaeaacqaIXaqmaeaacqWGMbGzdaWgaaWcbaGaem4CamhabeaaaaGccqGHsislcqaIXaqmaiaawIcacaGLPaaacqWGfbqrdaWgaaWcbaGaemyDauhabeaakiaaxMaacaWLjaWaaeWaaeaacqaIXaqmaiaawIcacaGLPaaaaaa@3E1F@

where *E*_*s *_and *E*_*u *_denote the average time for successful and unsuccessful runs, respectively, and *f*_*s *_is the fraction of successful runs [24]. Unsuccessful runs were terminated after 1800 CPU seconds for structures that are decomposed by RNA-SSD. For structures not subject to decomposition, the algorithm terminates in less time, determined by the maximal number *n*_*L *_of base modifications performed by the SLS procedure without finding a solution. In our experiments, we used *n*_*L *_= 1000. For structures with no successful runs, we arbitrarily reported the expected run time to be 10^6 ^CPU seconds.

For the experiments with primary structure constraints, 50 runs were performed for each structure and set of primary structure constraints. The expected CPU time required for designing a structure with a given constraint was estimated from these runs using the same formula as in the unconstrained case, and the median over the 50 sets of constraints per structure was used for all analyses.

(The data sets and the algorithm will be made available on-line at the time of publication.)

## Authors' contributions

RAH, HH and AC jointly developed the improvements and extensions of RNA-SSD, designed experiments and analysed results; RAH implemented the improved version of RNA-SSD and performed all experiments; all authors were involved in writing the paper.

## Appendix

**Figure 11:**
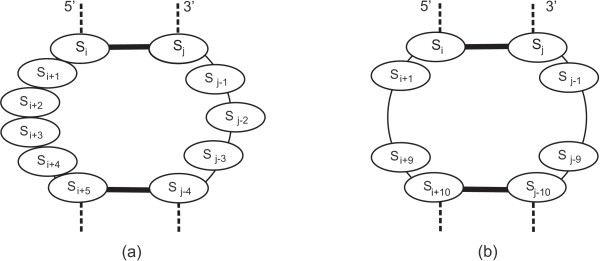
**Energetically favourable structures**. (a) Motif I: internal loop formed by breaking the base pair *s*_*i*+1_·*s*_*j*-3 _from motif B; (b) Motif 1I: internal loop formed by breaking the base pair *s*_*i*+6_·*s*_*j*-4 _from motif 2I.

Consider the structure motif B from Figure 6a, which is formed by two bulges of size two and three, respectively, where *s*_*i *_∈ {A, G, C, U} for all *i*. We will show that this structure is impossible to design with the standard thermodynamic model, because the internal loop I of size seven, formed by breaking the base pair *s*_*i*+1_·*s*_*j*-3 _(see Figure 11a), is energetically more favourable.

Let *S *be a set of assignments of bases in which the base pairs are complementary. Let Δ*G*(*B, s*) and Δ*G*(*I, s*) be the energies of motif *B *and *I*, respectively, for *s *∈ *S*. We will show that

Δ*G*(*I*, *s*) < Δ*G*(*B*, *s*) ∀*s *∈ *S*

following the notation of Andronescu [25] and using the thermodynamic parameters of Mathews et al. [4] for computing Δ*G*(*B*, *s*) and Δ*G*(*I*, *s*). Table 7 shows the definition of parameters and their possible values involved in the calculation of the free energy of an internal loop.

**Table 7:** Free energy parameters for internal loops.

Parameter	Explanation	Values
Δ*G-length-I*(*n*)	Destabilizing energy of internal loop of size *n *(*n *= 4, 5, 6,...)	1.7, 1.8, 2.0,...
Δ*G-length-B*(*n*)	Destabilizing energy of bulge of size *n *(*n *= 1, 2, 3,...)	3.8, 2.8, 3.2,...
Δ*G-Internal-n *(*s*_*i*_, *s*_*j*_, *s*_*i*+1_, *s*_*j*-1_)	Terminal mismatch free energy of closing base pair (*s*_*i*_·*s*_*j*_) and neighbouring free bases *s*_*i*+1 _and *s*_*j*-1_	-1.1, -0.7, -0.4, 0.0 and 0.7
Δ*G-Asymmetry*(*l*_1_, *l*_2_)	Penalty for asymmetric internal loops	min={30.5⋅|l1−l2| MathType@MTEF@5@5@+=feaafiart1ev1aaatCvAUfKttLearuWrP9MDH5MBPbIqV92AaeXatLxBI9gBaebbnrfifHhDYfgasaacH8akY=wiFfYdH8Gipec8Eeeu0xXdbba9frFj0=OqFfea0dXdd9vqai=hGuQ8kuc9pgc9s8qqaq=dirpe0xb9q8qiLsFr0=vr0=vr0dc8meaabaqaciaacaGaaeqabaqabeGadaaakeaaieGacqWFTbqBcqWFPbqAcqWFUbGBcqGH9aqpdaGabeqaauaabaqaceaaaeaacqaIZaWmaeaacqaIWaamcqGGUaGlcqaI1aqncqGHflY1daabdaqaaiabdYgaSnaaBaaaleaacqaIXaqmaeqaaOGaeyOeI0IaemiBaW2aaSbaaSqaaiabikdaYaqabaaakiaawEa7caGLiWoaaaaacaGL7baaaaa@4222@

Δ*G*(*I*, *s*) = Δ*G-length-I*(*7*) + Δ*G-Internal-n*(*s*_*i*_, *s*_*j*_, *s*_*i*+1_, *s*_*j*-1_)

+ Δ *G-Internal-n*(*s*_*i*+5_, *s*_*j*-4· _*s*_*i*+4_, *s*_*j*-3_)

+ Δ *G-Asymmetry*(4, 3) ∀*s *∈ *S*

⇒ *max*_*s*∈*S*_{Δ*G*(*I*, *s*)} = 2.7 + *max*_*s*∈*S*_{Δ*G-Internal-n*(*s*_*i*_, *s*_*j*_, *s*_*i*+1_, *s*_*j*-1_)

+ Δ *G-Internal-n*(*s*_*i*+5_, *s*_*j*-4_·*s*_*i*+4_, *s*_*j*-3_)}

= 2.7 + 2·07 = 4.1

On the other hand,

Δ*G*(*B*, *s*) = Δ*G-length-B*(2) + Δ*G-length-B*(3)

= 2.8 + 3.2 = 6 ∀*s *∈ *S*

Then

*max*_*s*∈*S*_{Δ*G*(*I, s*)} <*min*_*s*∈*S *_{Δ*G*(*B, s*)}.

Therefore,

Δ*G*(*I*, *s*) < Δ*G*(*B*, *s*) ∀*s *∈ *S*.

Consider the motif 2*I *in Figure 6b, which is formed by two internal loops both of size eight. We will show that this structure motif is impossible to design, because the internal loop 1*I *of size eighteen, formed by breaking the base pair *s*_*i*+6_·*s*_*j*-4 _(see Figure 11b), is more favourable. Let

Δ*G-Internal-n*(*s*_*i*_, *s*_*j*_, *s*_*i*+1_, *s*_*j*-1_) = *x*_1_

Δ*G-Internal-n*(*s*_*j*-10_, *s*_*i*+10_, *s*_*j*-9_, *s*_*i*+9_) = *x*_2_

Δ*G-Internal-n*(*s*_*j*-4_, *s*_*i*+6_, *s*_*j*-3_, *s*_*i*+5_) = *y*_1_

Δ*G-Internal-n*(*s*_*i*+6_, *s*_*i*-4_, *s*_*i*+7_, *s*_*j*-5_) = *y*_2_

where *x*_1_, *x*_2_, *y*_1_, *y*_2 _∈ {-1.1, -0.7, -0.4, 0, 0.7}. Then

Δ*G*(1*I*, *s*) = Δ*G-length-I*(18) + *x*_*1 *_+ *x*_2 _∀*s *∈ *S*

⇒ Δ*G*(1*I*, *s*) = 3.1 + *x*_1 _+ *x*_2 _∀*s *∈ *S*.

On the other hand,

Δ*G*(2*I*, *s*) = [Δ*G-length-I*(8) + *x*_1 _+ *y*_1 _+ Δ*G-Asymmetry*(3, 5)]

+ [Δ*G-length-I*(8) + *x*_2 _+ *y*_2 _+ Δ*G-Asymmetry*(3, 5)] ∀*s *∈ *S*

⇒ Δ*G*(2*I*, *s*) = (2.3 + *x*_1 _+ *y*_1 _+ 1) + (2.3 + *y*_2 _+ *x*_2 _+ 1) ∀*s *∈ *S*

⇒ Δ*G*(2*I*, *s*) = 6.6 + *x*_1 _+ *y*_1 _+ *y*_2 _+ *x*_2 _∀*s *∈ *S*.

Therefore,

Δ*G*(1*I*, *s*) < Δ*G*(2*I*, *s*) ∀*s *∈ *S*.
